# Developmental trauma in functional motor disorder: the mediating roles of affective symptoms and multimorbidity

**DOI:** 10.1017/S0033291726103821

**Published:** 2026-03-27

**Authors:** Petr Sojka, Martin Máčel, Lucia Nováková, Barbora Křupková, Tomáš Sieger, Tomáš Nikolai, Mark J. Edwards, Tereza Serranová

**Affiliations:** 1 Charles University First Faculty of Medicine: Univerzita Karlova 1 lekarska faku, Czech Republic; 2 Charles University Faculty of Arts: Univerzita Karlova Filozoficka fakulta, Czech Republic; 3 King’s College London Institute of Psychiatry Psychology & Neuroscience, United Kingdom; 4 General University Hospital in Prague: Vseobecna Fakultni Nemocnice v Praze, Czech Republic

**Keywords:** anxiety, depression, developmental trauma, functional motor disorder, multimorbidity, structural equation modeling

## Abstract

**Background:**

Childhood trauma is common in functional motor disorder (FMD), but it is unclear whether specific trauma dimensions are differentially linked to symptom burden, and whether depression, anxiety, or multimorbidity can mediate these associations.

**Methods:**

We conducted a cross-sectional case–control study including 322 patients with clinically definite FMD and 215 neurologically healthy controls, balanced with respect to age and sex. Six outcomes – motor symptom severity, cognitive complaints, depression, anxiety, fatigue, and pain – were jointly modeled using Bayesian multivariate regression with Childhood Trauma Questionnaire subscales as predictors. Bayesian structural equation modeling tested mediation by depression, anxiety, and multimorbidity.

**Results:**

In FMD, emotional abuse was the most consistent trauma correlate, associated with higher depression (β = 0.37, 95% CrI 0.22–0.51), anxiety (β = 0.32, 95% CrI 0.16–0.47), cognitive complaints (β = 0.27, 95% CrI 0.11–0.42), fatigue (β = 0.17, 95% CrI 0.03–0.32), and motor symptom severity (β = 0.15, 95% CrI 0.04–0.25). Mediation analyses indicated that affective symptoms fully accounted for trauma–symptom associations (indirect effect β = 0.42, 95% CrI 0.27–0.56). Multimorbidity was associated with more severe affective symptoms (β = 0.24, 95% CrI 0.12–0.37) and FMD symptoms (β = 0.24, 95% CrI 0.07–0.42) but did not mediate trauma–symptom relationships.

**Conclusions:**

Emotional abuse is a key developmental risk factor for FMD, with its effects on symptom severity mediated by depression and anxiety. Multimorbidity increases symptom burden but is not a primary pathway linking trauma to FMD. Findings support routine trauma and affective symptom screening in FMD and targeted psychotherapeutic interventions.

## Introduction

Functional neurological disorder (FND) is a common, disabling, and often persistent condition with phenotypically diverse manifestations affecting voluntary motor functions with associated non-motor and psychiatric symptoms (Anderson et al., 2007; Finkelstein, Diamond, Carson, & Stone, 2025; Gelauff, Stone, Edwards, & Carson, 2014). Historically, explanations for FND have centered on acute psychological conflicts or single-event trauma leading to symptom onset, consistent with classical psychodynamic models of conversion (Kanaan & Craig, 2019). Such stressors were long viewed as causal but are now recognized as neither necessary nor sufficient for FND development. Current evidence suggests a heterogeneous multifactorial etiology, with varying contributions from biological and psychosocial factors playing roles as predisposing, precipitating, and perpetuating factors. While the causal importance of single traumatic events has been revised, contemporary research increasingly implicates *developmental trauma* – chronic exposure to adverse childhood experiences (ACEs), such as emotional neglect, physical abuse, or family dysfunction – as a key contributor to the pathogenesis of neuropsychiatric disorders (Teicher, Gordon, & Nemeroff, 2022). This shift reflects a growing recognition that cumulative stress, rather than a single traumatic event, shapes long-term vulnerability to a chronic disease via a variety of biological, psychological, and social mechanisms, including by impairing the function of neural, immune, and endocrine systems (Guidi, Lucente, Sonino, & Fava, 2021).

ACEs refer to sustained or repeated exposures during childhood that represent deviations from an expectable caregiving environment and typically involve threat, deprivation, or both (Bhutta, Bhavnani, Betancourt, Tomlinson, & Patel, 2023). These experiences include active forms of maltreatment, such as emotional, physical, and sexual abuse, as well as passive forms such as emotional and physical neglect. ACEs also encompass broader household and environmental adversities, including witnessing domestic violence, parental mental illness or substance use, institutional care, family separation, and chronic socioeconomic deprivation (Bhutta et al., 2023). From a developmental perspective, ACEs are thought to influence vulnerability through enduring effects on affect regulation, stress responsivity, and self-related processing (Nelson, Sullivan, & Valdes, 2025). Exposure to chronic maltreatment during sensitive developmental windows has been linked to enduring alterations in hypothalamic–pituitary–adrenal axis functioning, autonomic regulation, immune and inflammatory processes, and experience-dependent maturation of frontolimbic, salience, and self-referential neural networks (Nelson et al., 2025). These alterations are not disorder-specific but may create a general vulnerability that shapes how later stressors are processed (Gee, 2021). In the context of FND, ACEs are therefore increasingly conceptualized as predisposing developmental risk factors that interact with affective, cognitive, and bodily processes to influence symptom expression and persistence (Hallett et al., 2022).

Patients with FND report significantly more adverse life experiences and maltreatment across the lifespan than both healthy and psychiatric controls, with a meta-analysis showing these events occur twice as often as in other psychiatric conditions and eight times as often as in healthy individuals (Ludwig et al., 2018). In functional motor disorder (FMD), the most common subtype of FND, a key gap remains in understanding how specific types of childhood adversity contribute to the clinical heterogeneity of the disorder. Prior work has often relied on small mixed-phenotype FND samples (e.g. including functional seizures) and cumulative risk models, obscuring how specific forms of childhood trauma (e.g. emotional neglect versus physical abuse) differentially shape clinical outcomes (Gee, 2021). Only a few studies have directly mapped distinct trauma dimensions onto FMD symptom severity, but these have used a somatoform dissociation measure that has poor specificity for FND symptoms (Roelofs, Keijsers, Hoogduin, Näring, & Moene, 2002; Roelofs, Spinhoven, Sandijck, Moene, & Hoogduin, 2005; Spinhoven et al., 2004). Given the broad spectrum of FMD presentations, including different motor phenotypes and numerous non-motor symptoms, such as pain, fatigue, cognitive, and affective symptoms that often coexist in one individual, identifying trauma-specific symptom patterns could help refine etiological models and inform individualized treatment approaches.

The relationship between ACEs and FMD symptoms may unfold along distinct trajectories, with affective symptoms and multimorbidity emerging as two potential mediating pathways. First, patients with FMD consistently report high rates of psychiatric comorbidities, most commonly affective disorders such as depression and anxiety (Butler et al., 2021; Feinstein, Stergiopoulos, Fine, & Lang, 2001; Gelauff et al., 2014; Macchi, Kletenik, Olvera, & Holden, 2021), which are, however, also considered risk factors for developing FMD (Hallett et al., 2022). Depression and anxiety show substantial overlap with FMD in terms of shared symptoms (e.g. fatigue, dizziness, cognitive difficulties, and sleep disturbances), common age of onset (typically in the early 20s to midlife), underlying neurobiological features (e.g. altered limbic circuitry; Perez et al., 2021), and associations with early life adversity (Lian, Kiely, Callaghan, & Anstey, 2024). This raises the possibility that ACEs contribute to emotional dysregulation, which in turn exacerbates functional neurological symptoms, rather than exerting direct effects. However, it is equally plausible that depression and anxiety arise as downstream effects of living with a disabling condition like FMD, rather than serving as intermediaries (Gendre et al., 2019).

The second possible trajectory builds on an increasing recognition that FMD rarely occurs in isolation (Butler et al., 2021). Many individuals experience co-existence of multiple chronic conditions (i.e. multimorbidity), including chronic pain, migraine, cerebrovascular disease, and autoimmune conditions prior to FMD diagnosis (Ducroizet et al., 2023; Tinazzi et al., 2021). The cumulative impact of prolonged stress and adverse life experiences may contribute to the development of a broad spectrum of chronic health conditions (Guidi et al., 2021), potentially shaping vulnerability to FMD (Keynejad et al., 2020). However, whether multimorbidity represents a pathway through which trauma influences FMD symptomatology remains unknown.

Given these gaps in the existing literature, we hypothesized that different dimensions of childhood trauma would show non-uniform associations with motor and non-motor symptom domains, rather than exerting equivalent effects across outcomes. Second, we hypothesized that affective symptoms (depression and anxiety) would constitute a key pathway linking childhood trauma to functional symptom severity, consistent with models emphasizing emotional dysregulation as a central mechanism. Third, we hypothesized that multimorbidity would represent an additional candidate pathway linking childhood trauma to symptom burden, reflecting broader health vulnerability and disease accumulation.

To disentangle the pathways linking early-life adversity to symptom expression in FMD, analytic approaches are needed that move beyond simple group comparisons or bivariate associations. Multivariate approaches provide a principled framework to map adversity dimensions on multiple symptom expressions and test whether childhood trauma influences FMD symptoms directly or indirectly through affective symptoms or multimorbidity. This approach is especially suitable for FMD, given its clinical heterogeneity and the overlapping symptom dimensions. Accordingly, the present case–control study applies Bayesian multivariate regression and structural equation modeling in a well-characterized cohort of patients with FMD (N = 322) to identify key pathways through which developmental adversity shapes FMD presentations.

## Methods

### Participants

Three-hundred twenty-two consecutive outpatients from a specialized FND clinic diagnosed with clinically definite FMD according to Gupta and Lang criteria participated in the study. Two-hundred fifteen controls with no history of neurological disease of the central or peripheral nervous system were recruited from a local database of healthy volunteers and through community advertisements and outreach. Controls were included after performing a medical history and verifying a normal neurological examination. Exclusion criteria included age < 18 years old and intellectual disability, psychiatric condition, or physical impairment that precluded the provision of informed consent. Controls were recruited with the aim of group-balancing patients on sex and age. The study was approved by the General University Hospital in Prague ethics committee (approval no. 37/19).

### Instruments

Childhood trauma was assessed retrospectively using the 28-item Childhood Trauma Questionnaire (CTQ) (Bernstein et al., 1994), which yields five subscale scores for emotional abuse, emotional neglect, physical abuse, physical neglect, and sexual abuse; higher scores indicate greater exposure. The presence of trauma was defined according to the clinical cut-off thresholds established by Walker et al. (1999).

Motor and non-motor symptom burden was assessed across multiple domains.

All patients evaluated their own motor symptom severity on a 3-point Likert scale (not bothered at all = 0, bothered a little = 1, bothered a lot = 2) according to the adapted version of Patient-Health-Questionnaire (PHQ-15). In addition to PHQ-15 items assessing motor function, including weakness, motor coordination impairment, and gait disorder, we added one item assessing tremor and jerks and one item assessing abnormal postures or spasms. The total score (subjective motor symptoms severity [SMSS], range 0–10) was calculated (Forejtová et al., 2023).

Affective symptoms were measured with the Beck Depression Inventory-II (BDI-II) (Beck, Steer, & Brown, 2011), assessing depressive symptoms over the previous 2 weeks, and the trait subscale of the State–Trait Anxiety Inventory (STAI-Y2) (Spielberger, 1983).

Cognitive complaints were captured using the Czech adaptation of the Cognitive Complaints Questionnaire (QPC) (Markova et al., 2017). Fatigue was evaluated with the Fatigue Severity Scale (FSS) (Krupp, LaRocca, Muir-Nash, & Steinberg, 1989), and pain with the PainDetect visual analogue scales (Freynhagen, Baron, Gockel, & Tölle, 2006) for current, average, and maximum pain in the previous 4 weeks; average of these values (0–10) was used for analysis.

Higher scores on all measures indicate greater symptom severity.

To assess the combined effects of several coexisting diseases, a multimorbidity index (MMi) was calculated as the sum of major physical, neurological, and psychiatric conditions present in each patient. Psychiatric comorbidity was coded as one point if any psychiatric diagnosis (e.g. depression, anxiety, PTSD, or personality disorder) was present to avoid inflating the index with overlapping mood/anxiety diagnoses. Data from self-reports and medical reports were used to calculate the MMi. The selection of illnesses was based on the most prevalent physical and neurological diseases (Diederichs, Berger, & Bartels, 2011). The whole list of illnesses can be seen in Table 1.

**Table 1.** The multimorbidity indices

Physical illnesses	Neurological illnesses	Psychiatric illnesses
Hypertension (24%)	Radicular pain (17%)	Major depressive disorder (13%)
Diabetes mellitus (7%)	Polyneuropathy (5%)	Anxiety disorder (11%)
Cardiovascular disease (11%)	Infectious disease (2%)	Anxiety-depressive disorder (38%)
Dyslipidemia (14%)	Essential tremor (3%)	Phobia (7%)
Thyroid disorders (23%)	Parkinson’s disease (1%)	Panic disorder (6%)
Asthma (17%)	Dystonia (3%)	PTSD (17%)
GIT disorder (17%)	Stroke (4%)	Personality disorder (2%)
Rheumatic disorders or arthrosis (16%)	Demyelinating disease (1%)	OCD (2%)
Autoimmune disease (4%)	Autoimmune disease (1%)	Other (2%)
Gynecological and reproductive disorders (9%)	Neuromuscular (3%)	
Oncological disease (2%)	Epilepsy (2%)	
Migraine (18%)	Other (8%)	
Obesity (11%)		
Other (17%)		

All illnesses were summed except for psychiatric illnesses where having at least one illness was counted as a comorbidity. Percentages show occurrence of illnesses in the FMD sample. GIT = gastrointestinal tract; PTSD = post-traumatic stress disorder; OCD = obsessive-compulsive disorder.

Data collection was conducted primarily in person during study visits. Questionnaire data were completed on a desktop computer using the REDCap (Research Electronic Data Capture) platform; when participants were unable to complete questionnaires electronically, data were collected using paper-and-pencil formats and subsequently entered into REDCap by study staff.

### Analysis

Using a Bayesian multivariate regression, we jointly modeled six outcomes: SMSS, subjective cognitive symptoms (QPC), depression (BDI-II), anxiety (STAI-Y2), average pain (PainDetect), and fatigue (FSS). Predictors were the five CTQ subscales, age and group (FMD vs healthy controls), with group × CTQ interactions to test for differential associations. All numeric variables were z-standardized (mean = 0, SD = 1). To ensure stable and conservative estimation in the presence of correlated predictors and outcomes, we used regularized horseshoe priors on regression coefficients and an LKJ(2) prior on the residual correlation matrix. These priors reduce overfitting, improve robustness in multivariate models, and are well suited to detecting sparse trauma–symptom associations. Outcomes were modeled using a multivariate Student-t distribution to account for skew and heavy-tailed clinical and trauma scores. Models were run with 4 chains × 5000 iterations (1000 warm-up). Convergence was confirmed by R̂ ≈ 1.00 and sufficient effective sample sizes. Results are reported as posterior means with 95% credible intervals; Bayesian R^2^ was computed for each outcome. Because variables were standardized, regression coefficients are expressed in SD units.

To test whether the associations between CTQ dimensions and FMD symptoms were mediated by anxiety/depression or by multimorbidity, we used Bayesian Structural Equation Modeling (BSEM) implemented in *blavaan.* BSEM combines Bayesian confirmatory factor analysis with Bayesian regression, allowing simultaneous modeling of latent constructs and their directional associations. Three latent variables were specified: (i) CTQ, indicated by the five CTQ subscales; (ii) DEPANX, indicated by BDI-II and STAI-Y2; and (iii) FMD, indicated by SMSS, QPC, FSS, and average pain. Structural paths tested whether the effect of CTQ on FMD was mediated by DEPANX and/or multimorbidity. Factor loadings for the manifest indicators are reported in Supplementary Table 1. Blavaan’s default weakly informative priors were used for factor loadings and latent variable variances. Model fit was evaluated using Bayesian Comparative Fit Index (BCFI), Bayesian Tucker- Lewis Index (BTLI), and Bayesian Root Mean Square Error of Approximation (BRMSEA).

To address the potential overlap of depressive/anxious symptoms (DEPANX) with non-motor symptoms within the FMD factor (e.g. fatigue and subjective cognitive complaints), we performed sensitivity analyses in which only SMSS (motor symptoms) were retained in the FMD factor. Similarly, for MMi, we conducted a secondary analysis excluding psychiatric illness, retaining only somatic and neurological illnesses.

Group comparisons were conducted using Bayesian t-tests. To compare trauma prevalence between groups, we used Bayesian logistic regression.

All statistical analyses were conducted within a Bayesian framework; accordingly, results are reported using posterior estimates with 95% credible intervals and Bayes factors (BF₁₀).

Analyses were performed in R (Version 4.4, R Core Team, 2024) using *brms* (Bürkner, 2017) and *blavaan* (Merkle, Fitzsimmons, Uanhoro, & Goodrich, 2021) packages, path diagrams were made using the *semPlot* package (Epskamp, 2013).

## Results

### Descriptives

The FMD group comprised 322 individuals (73.6% female; mean age 48.8 years, SD 12.4), and the control group included 215 participants (74.0% female; mean age 44.7 years, SD 12.2). Groups did not differ in sex distribution, whereas patients with FMD were on average older (mean difference 4.05 years, 95% CrI 1.92 to 6.18). Across all symptom domains, patients with FMD reported markedly greater burden than controls, including higher scores for motor symptom severity, depression, anxiety, fatigue, pain, and subjective cognitive complaints. Childhood trauma exposure was also consistently higher in FMD across all CTQ subscales. Bayesian logistic regression indicated that the odds of reporting no trauma were approximately twice as high in the control group relative to the FMD group. The average MMi in the FMD group was 3.3 (SD 1.8), reflecting the presence of multiple co-existing medical and psychiatric conditions. Within the FMD cohort, gait disorder was the most frequent motor phenotype, followed by tremor and weakness. No meaningful differences in trauma or symptom measures were observed among motor phenotypes. Sociodemographic and clinical characteristics are summarized in Supplementary Table 2.

### Associations between CTQ dimensions and FMD symptoms

**Figure 1.**
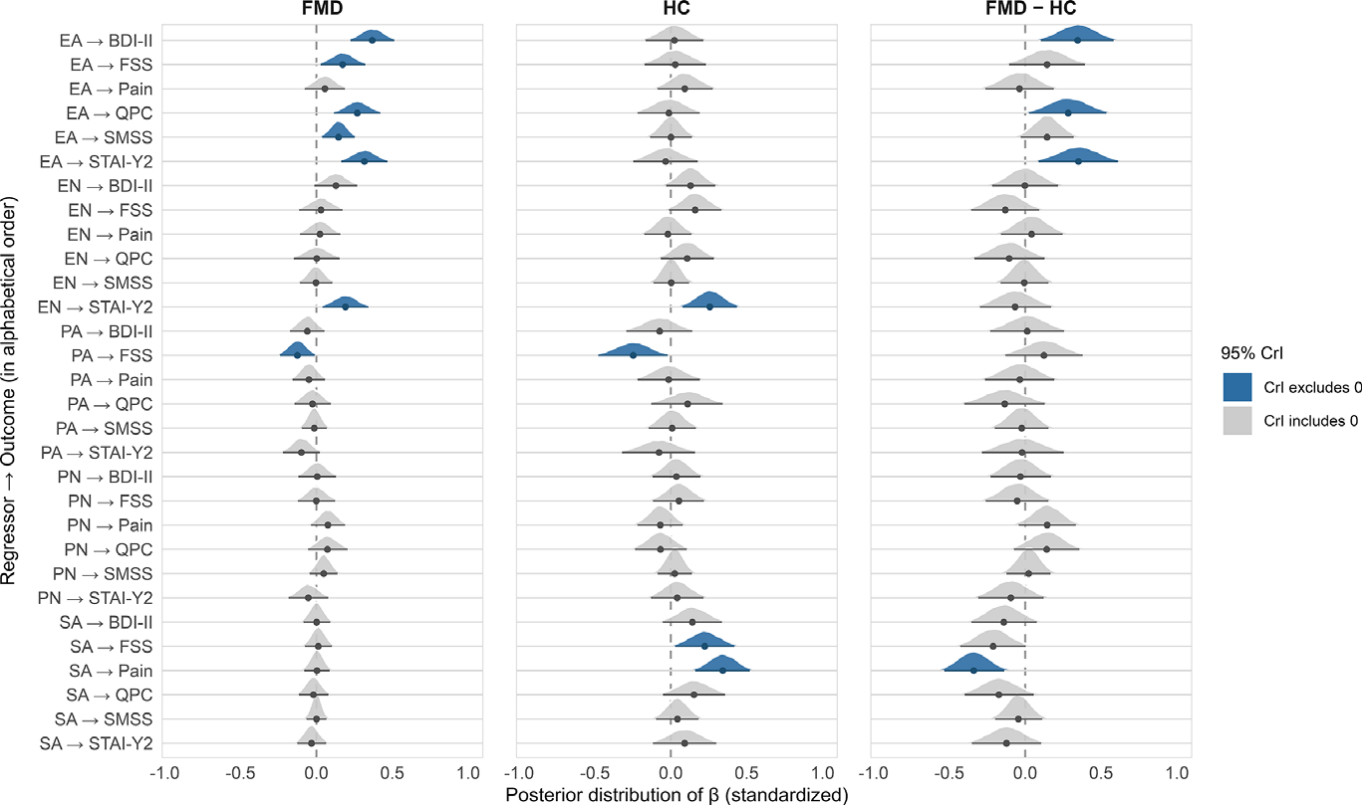
Associations between Childhood Trauma Questionnaire (CTQ) subscales and symptom outcomes in functional motor disorder (FMD) vs controls. Posterior distributions of standardized regression coefficients from the Bayesian multivariate model for each CTQ subscale (EA = emotional abuse; EN = emotional neglect; PA = physical abuse; PN = physical neglect; SA = sexual abuse) predicting SMSS (motor symptoms), BDI-II (depression), STAI-Y2 (anxiety), FSS (fatigue), Pain (average), and QPC (subjective cognitive complaints). Estimates are shown separately for the FMD and control group; the middle panel depicts differences in slopes between the FMD and control group. Positive values indicate higher symptom burden with higher trauma exposure. Error bars denote 95% credible intervals. All β coefficients represent standardized slopes, that is, the expected change (in SD units) in a given symptom outcome associated with a 1 SD increase in the predictor.

Across trauma dimensions, emotional abuse emerged as the most consistent predictor of symptom burden in FMD, showing positive associations with motor, affective, subjective cognitive symptoms, and fatigue. These associations were attenuated in HC, while sexual abuse was more strongly linked to pain and fatigue in HC. Additionally, emotional neglect was linked with higher anxiety, whereas physical abuse was negatively associated with fatigue in both groups. Other trauma subscales showed only minor effects. For full posterior distributions of standardized regression coefficients in FMD, HC, and their differences, see Figure 1, and for mean values and 95% credible intervals of standardized regression coefficients, see Supplementary Table 3.

Model performance was acceptable across outcomes: Bayesian R^2^ indicated good explanatory power for motor symptoms (R^2^ ≈ 0.66) and pain (R^2^ ≈ 0.49), with moderate fit for depression, fatigue, subjective cognitive complaints (R^2^ ≈ 0.35–0.40), and anxiety (R^2^ ≈ 0.34). All models converged adequately (R̂ = 1.00, effective sample sizes >6,000). Residual correlations were moderate-to-high, most notably between depression and anxiety (ρ ≈ 0.77) and between depression and subjective cognitive complaints (ρ ≈ 0.53), consistent with shared affective variance.

### Affective symptoms as mediators of trauma-FMD symptoms

**Figure 2.**
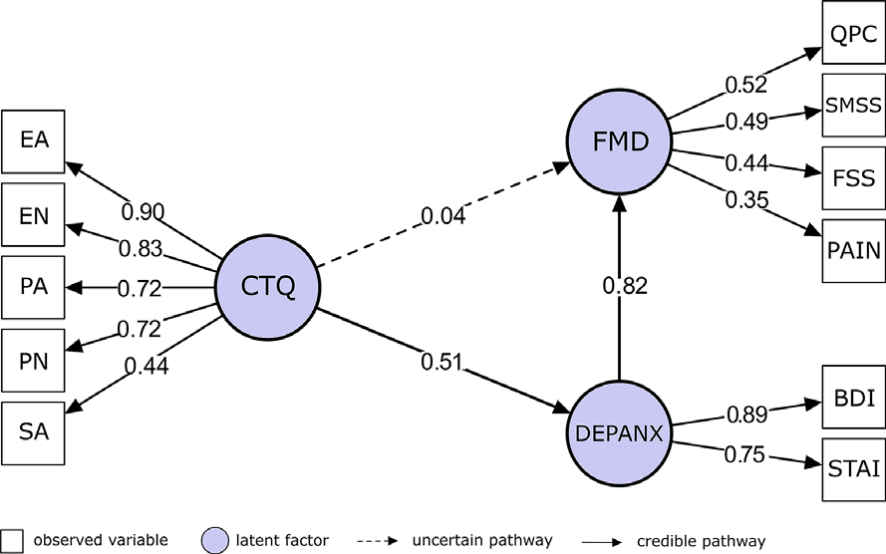
Mediation of the Childhood Trauma Questionnaire (CTQ) → functional movement disorder (FMD) association by affective symptoms. Bayesian structural equation model illustrating indirect and direct pathways between childhood trauma and FMD symptom burden. The latent variable CTQ represents the five Childhood Trauma Questionnaire subscales: EA = emotional abuse, EN = emotional neglect, PA = physical abuse, PN = physical neglect, and SA = sexual abuse. The latent variable DEPANX captures affective symptoms, indexed by the Beck Depression Inventory–II (BDI-II) and the State–Trait Anxiety Inventory – Trait form (STAI). The latent variable FMD reflects functional symptom severity, measured by the Subjective Motor Symptom Severity Scale (SMSS), Cognitive Complaints Questionnaire (QPC), Fatigue Severity Scale (FSS), and Pain. Standardized path coefficients are shown adjacent to arrows. The direct path from CTQ to FMD is negligible, whereas the indirect path via DEPANX is robust, indicating full mediation by affective symptoms.

**Figure 3.**
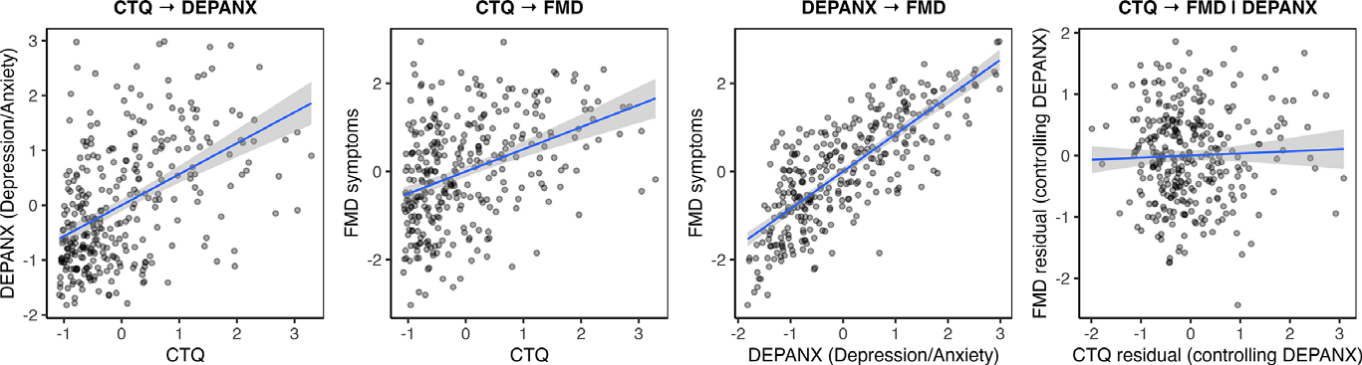
Associations between the factors in the mediation model. The latent variable CTQ represents the five Childhood Trauma Questionnaire subscales. The latent variable DEPANX captures affective symptoms, indexed by the Beck Depression Inventory–II and the State–Trait Anxiety Inventory – Trait form. The latent variable FMD reflects functional symptom severity, measured by the Subjective Motor Symptom Severity Scale, Cognitive Complaints Questionnaire, Fatigue Severity Scale, and Pain.

Bayesian structural equation modeling indicated that affective symptoms (DEPANX, estimated as a linear combination of 0.89·BDI + 0.75·STAI-Y) fully mediated the association between childhood trauma (CTQ) and FMD symptoms, meaning that childhood trauma was related to FMD symptoms only through its effect on depression/anxiety symptoms. In the mediation model, the direct effect of CTQ on FMD symptoms was negligible (β ≈ 0.04, 95% CrI [−0.14, 0.20]), whereas the indirect effect via DEPANX was robust (β ≈ 0.42, 95% CrI [0.27, 0.56]). DEPANX strongly predicted FMD symptoms (β ≈ 0.82, 95% CrI [0.62, 1.03]), and CTQ remained a strong predictor of DEPANX (β ≈ 0.51, 95% CrI [0.38, 0.64]). See Figure 2 for the mediation model and Figure 3 for associations between the factors. The full Bayesian parameter estimates are in Supplementary Table 4A.

In the initial model without the mediator, CTQ was positively associated with both DEPANX (β ≈ 0.51, 95% CrI [0.37, 0.65]) and FMD symptoms (β ≈ 0.35, 95% CrI [0.20, 0.50]).

The mediation model explained 47% of the variance in FMD symptoms and 20% of the variance in DEPANX. Bayesian fit indices indicated acceptable model fit (BCFI ≈ 0.95, 95% CrI [0.94, 0.96], BTLI ≈ 0.91, 95% CrI [0.89, 0.93], BRMSEA ≈ 0.09, 95% CrI [0.09–0.10]). All model parameters showed adequate convergence (R̂ = 1.00).

A sensitivity analysis retaining only SMSS as the sole indicator of the FMD latent construct yielded consistent results, further supporting the stability of the mediation pathway. The association between CTQ and motor symptom severity remained fully mediated by DEPANX, with no meaningful direct effect of CTQ on SMSS. For full results of the reduced model, see Supplementary Table 4B.

We also tested the reverse model, in which FMD symptoms served as the mediator between CTQ and DEPANX. Although FMD symptoms predicted DEPANX (β ≈ 0.83, 95% CrI [0.63, 1.05]), the direct effect of CTQ on DEPANX (β ≈ 0.37, 95% CrI [0.22, 0.52]) exceeded the indirect effect via FMD symptoms (β ≈ 0.31, 95% CrI [0.17, 0.45]), indicating the absence of meaningful mediation. These findings therefore support DEPANX as the primary pathway linking ACEs to FMD symptom burden. Full results are presented in Supplementary Table 5.

### Multimorbidity as a mediator of trauma-FMD symptoms

Including multimorbidity (MMi) in the mediation model did not alter the primary pathway linking ACEs to FMD symptoms via affective symptoms. The direct effect of CTQ on FMD symptoms remained negligible (β ≈ 0.03, 95% CrI [−0.14, 0.21]), while the indirect effect through affective symptoms remained strong (β ≈ 0.38, 95% CrI [0.25, 0.51]). CTQ continued to strongly predict affective symptoms (β ≈ 0.49, 95% CrI [0.36, 0.63]).

**Figure 4.**
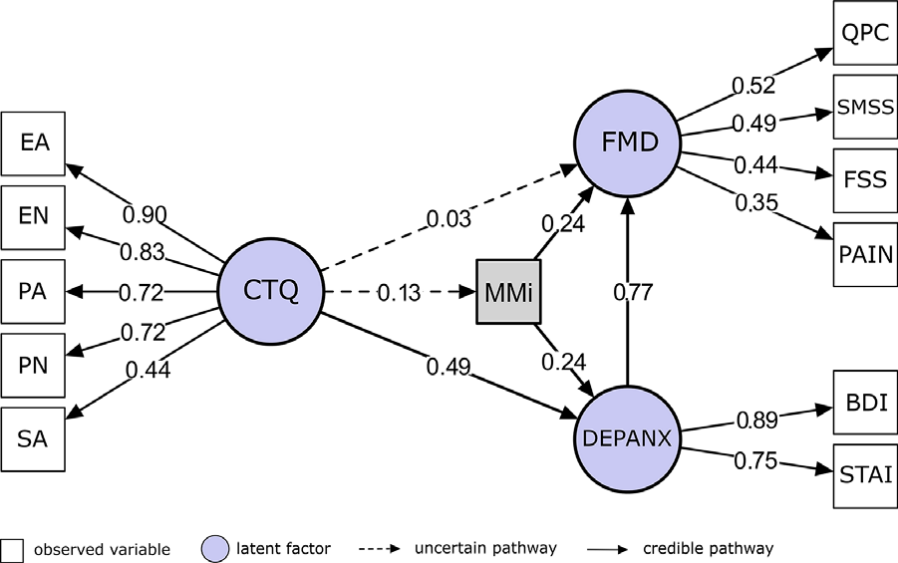
Mediation model including multimorbidity. Expanded Bayesian structural equation model incorporating multimorbidity (MMi) – defined as the total count of co-occurring medical, neurological, and psychiatric conditions – as an additional mediator of the relationship between childhood trauma and symptom severity in functional movement disorder (FMD). The latent variable Childhood Trauma Questionnaire (CTQ) represents five forms of childhood adversity: emotional abuse (EA), emotional neglect (EN), physical abuse (PA), physical neglect (PN), and sexual abuse (SA). The latent variable Affective Symptoms (DEPANX) is indicated by the Beck Depression Inventory–II (BDI) and the State–Trait Anxiety Inventory – Trait form (STAI). The latent variable Functional Movement Disorder Symptom Severity (FMD) is represented by the Subjective Motor Symptom Severity Scale (SMSS), Cognitive Complaints Questionnaire (QPC), Fatigue Severity Scale (FSS), and the Pain (average). The model shows that higher CTQ scores have small effects on MMi; MMi is associated with both affective symptoms and FMD severity but does not mediate the CTQ → FMD association. The principal pathway linking childhood trauma to FMD symptoms operates through affective symptoms (CTQ → DEPANX → FMD). Standardized path coefficients are shown adjacent to arrows.

MMi was positively associated with affective symptoms (β ≈ 0.24, 95% CrI [0.12, 0.37]) and with FMD symptoms (β ≈ 0.24, 95% CrI [0.07, 0.42]). The direct effect of ACEs on MMi was small (β ≈ 0.13, 95% CrI [0.002, 0.25]), explaining the negligible indirect effects of ACEs on FMD via MMi alone (β ≈ 0.03, 95% CrI [−0.01, 0.07]) or via MMi → affective symptoms (β ≈ 0.05, 95% CrI [−0.03, 0.1]). However, MMi exerted a small indirect effect on FMD symptoms through affective symptoms (β ≈ 0.18, 95% CrI [0.08, 0.29]). See Figure 4 depicting the mediation model with multimorbidity and Supplementary Table 6A for the full Bayesian parameter estimates.

The revised model explained 50% of the variance in FMD symptoms and 25% in affective symptoms, with acceptable Bayesian fit indices (BCFI ≈ 0.94, 95% CrI [0.93, 0.95], BTLI ≈ 0.91, 95% CrI [0.89, 0.93], BRMSEA ≈ 0.09 [0.08–0.1]). Convergence was satisfactory, with all R̂ values equal to 1.00.

When psychiatric diagnoses were excluded from the MMi, the revised index continued to predict greater FMD symptom severity but no longer related to affective symptoms. This suggests that the association between multimorbidity and affective symptoms was driven specifically by psychiatric comorbidity, whereas affective symptoms remained the primary pathway linking childhood trauma to FMD. Full results of the reduced model are provided in Supplementary Table 6B.

Moreover, we re-estimated the mediation model using an alternative MMi in which psychiatric comorbidities were summed rather than binarized. The pattern of results was similar to the original model, with negligible differences in regression coefficients; the largest change was in the association between multimorbidity and FMD symptom severity, which decreased by 0.037 points, from 0.245 [0.082, 0.415] to 0.208 [0.047, 0.386]. These results indicate that the coding of psychiatric comorbidity would not affect the primary findings.

## Discussion

In this large case–control study mapping the effects of different dimensions of childhood trauma on motor and non-motor symptoms in people with FMD, emotional abuse emerged as the trauma dimension most strongly associated with symptom burden. In controls, the trauma–symptom associations were attenuated except the link between sexual abuse and pain, as well as fatigue, that was stronger in control subjects. In FMD patients, the relationship between childhood trauma and FMD symptoms was fully explained by the pathway through anxiety and depression, with no direct effect once affective symptoms were included in the model. Multimorbidity contributed independently to symptom severity but did not mediate trauma-symptom associations. These findings refine prior work by showing, for the first time in a large cohort of patients with the motor subtype of FND, that specific trauma dimensions differ in their associations with motor and non-motor symptom burden and that these effects operate primarily through affective symptoms.

Our finding that emotional abuse was the most consistent correlate supports the role of emotional maltreatment in FMD symptoms reported in previous studies (Ludwig et al., 2018; Sar, Islam, & Oztürk, 2009) but differs from the meta-analysis by Ludwig et al. (2018), which emphasized emotional neglect. That review, however, was dominated by functional seizure cohorts and smaller, mixed-phenotype FND samples, whereas our work focuses exclusively on clinically definite FMD in a large, well-characterized cohort. Notably, sexual abuse was associated with pain in healthy controls but showed no specific symptom associations in FMD, which is interesting given the central role proposed for sexual trauma in the earlier accounts of the pathophysiology of FMD. Emotional abuse showed the strongest associations with the symptoms of depression and anxiety specifically in FMD, consistent with prior reports linking emotional maltreatment to severity of post-traumatic stress symptoms and altered stress responsivity in FND (Kienle et al., 2017; Weber et al., 2023). This is also in line with trauma research in non-FND populations showing that emotional abuse has particularly strong effects on frontolimbic networks and threat appraisal systems (Teicher & Samson, 2016).

Emotional abuse represents a chronic and identity-disruptive form of maltreatment. Unlike isolated physical or sexual assaults, it typically unfolds over years through repeated rejection, humiliation, or devaluation by caregivers and others. This persistent invalidation erodes a child’s developing sense of self, leaving enduring vulnerabilities in self-evaluation and affect regulation (Schlensog-Schuster et al., 2024; Teicher et al., 2022). Emotional abuse also amplifies the psychological impact of other maltreatment types, shaping the context in which episodic trauma is experienced (Edwards, Holden, Felitti, & Anda, 2003). Neuroimaging studies have linked traumatic experiences to cortical thinning and functional alterations in the precuneus and cingulate cortex – regions central to self-reflection, self-awareness, and emotional control (Heim, Mayberg, Mletzko, Nemeroff, & Pruessner, 2013; Wan, Rolls, Feng, & Cheng, 2022). In FMD, such disruptions in self-concept and affective processing may manifest as fearful attachment patterns – negative representations of self and others that contribute to anxiety, interpersonal mistrust, and heightened emotional reactivity (Williams, Ospina, Jalilianhasanpour, Fricchione, & Perez, 2019). Functional imaging evidence further supports the involvement of self-referential networks in FMD: reduced activation in the posterior cingulate and precuneus when attending to affected body parts (Spagnolo, Parker, Hallett, & Horovitz, 2025), and abnormal precuneus–temporoparietal junction connectivity correlated with motor symptom severity (Mueller et al., 2022), suggest that altered self-awareness may be an important factor in symptom generation.

The full mediation by affective symptoms supports mechanistic models in which early-life adversity fosters emotional dysregulation, which in turn exacerbates functional symptoms (Keynejad et al., 2019). Our finding that the reverse mediation model (FMD → affective symptoms) was non-significant strengthens the argument for affective processes as a key pathway. This interpretation aligns with evidence that emotional maltreatment produces lasting alterations in stress-regulatory systems, including blunted cortisol responsivity observed in emotional maltreatment-exposed adults (Carpenter et al., 2009), and with some FND-specific findings of lower basal cortisol levels and dysregulated autonomic reactivity linked to trauma burden (Apazoglou, Mazzola, Wegrzyk, Frasca, & Aybek, 2017; Weber et al., 2023). However, research on the biological impact of chronic stress in FND remains inconsistent, likely reflecting methodological heterogeneity across studies (Paredes-Echeverri et al., 2021). Our findings suggest that trauma-related alterations in affective processes, rather than trauma exposure per se, are the most proximal drivers of symptom burden.

Although multimorbidity did not mediate trauma–symptom associations, it exerted an independent effect on both affective and functional symptom burden. Notably, when psychiatric illnesses were removed, the revised MMi index predicted FMD symptoms specifically, but not affective burden. This suggests that the accumulation of chronic health conditions may reflect a broader vulnerability – potentially related to allostatic load or heightened symptom monitoring – rather than a distinct pathway from trauma to FMD. Individuals with higher multimorbidity may experience increased health-related anxiety (Ronaldson et al., 2021) and greater attentional focus on bodily sensations (Willadsen et al., 2021), all of which can reinforce functional symptom expression. Thus, while not a mechanistic bridge between trauma and FMD, multimorbidity remains clinically relevant as a contextual factor that intensifies symptom experience and healthcare utilization.

Routine screening for trauma history and affective symptoms should be considered in FMD assessment, given their role in symptom expression. Early, targeted psychotherapeutic interventions addressing emotional dysregulation – such as trauma-focused psychotherapy (Putica, Agathos, & Felmingham, 2025) or physiotherapy combined with CBT – may reduce both motor and non-motor symptom burden (Macías-García et al., 2024). As we found no differences in clinical or trauma variables across motor phenotypes, our results support refining FND subtyping to incorporate psychological profiles alongside broad phenotypic characteristics (Lidstone et al., 2022). Identifying subgroups based on trauma exposure and affective symptom profiles may facilitate personalized interventions and improve outcome prediction (Teicher & Samson, 2013). Future research should test these mediation pathways longitudinally, examine whether intervention on affective symptoms modifies motor outcomes, and explore potential neurobiological mediators linking trauma, affect, and motor dysfunction.

Limitations include retrospective trauma assessment, lack of trauma timing data, and the cross-sectional design, which precludes causal inference. Psychiatric comorbidities were based on self-report and medical records. Although a semi-structured diagnostic interview focusing on anxiety and depressive symptoms was conducted by a neurologist, no comprehensive, psychiatrist-led systematic assessment covering the full spectrum of psychopathology was performed. Consequently, neurodevelopmental conditions such as autism spectrum disorder and attention deficit hyperactivity disorder – known to be under-diagnosed in adult clinical settings – are likely under-reported in our sample. Although self-reported childhood adversity can be influenced by recall or disclosure biases, evidence suggests such biases typically underestimate rather than inflate associations (Hardt & Rutter, 2004). Emotional abuse is more frequently endorsed in retrospective self-report measures than some other trauma types, raising the possibility that its prominence reflects differential reporting rather than stronger causal relevance. However, because trauma dimensions were standardized and modeled simultaneously with regularization, the observed effects reflect relative association strength rather than prevalence. Moreover, the selective emergence of other trauma dimensions for specific outcomes argues against a simple reporting-frequency explanation. Emotional abuse may instead index chronic relational adversity that is particularly relevant to affective dysregulation and functional symptom expression. While our modeling approach allows testing of theoretically informed pathways, it does not establish causal mechanisms. Rather, the findings constrain plausible psychological interpretations by identifying which pathways are consistent with the observed multivariate symptom structure and which are not. Longitudinal and experimental studies will be required to directly test causal and biological mechanisms. Moreover, patients with FMD were on average older than controls, likely reflecting the control inclusion criteria requiring the absence of medical conditions in controls, which disproportionately excludes older individuals. Importantly, age was explicitly controlled for in the primary group × trauma interaction model, and all mediation analyses were conducted within the patient group only, such that age differences between patients and controls do not affect the main findings. Strengths of this study include a large, FMD-specific sample, comprehensive symptom characterization, and the use of Bayesian latent-variable models that account for measurement error and interdependence among symptom domains.

In conclusion, emotional maltreatment appears to be an important developmental risk factor for FMD, influencing symptom burden primarily through depression and anxiety. Multimorbidity contributes to illness severity but represents an independent contextual factor rather than a mediating mechanism. These findings support a developmental trauma–informed framework for understanding and managing FMD.

## Supporting information

10.1017/S0033291726103821.sm001 Sojka et al. supplementary material Sojka et al. supplementary material

## Data Availability

Data generated or analyzed during the study are available from the corresponding author by reasonable request.

## Abbreviations

ACEs: Adverse Childhood Experiences
BCFI: Bayesian Comparative Fit Index
BDI-II: Beck Depression Inventory-II
CrI: Credible Interval
CTQ: Childhood Trauma Questionnaire
DEPANX: Depression and Anxiety (latent variable)
FMD: Functional Motor Disorder
FND: Functional Neurological Disorder
PHQ: Patient Health Questionnaire
FSS: Fatigue Severity Scale
MMi: Multimorbidity Index
PHQ: Patient Health Questionnaire
QPC: Cognitive Complaints Questionnaire
BRMSEA: Bayesian Root Mean Square Error of Approximation
SEM: Structural Equation Modeling
SMSS: Subjective Motor Symptom Severity
SRMR: Standardized Root Mean Square Residual
STAI-Y2: State–Trait Anxiety Inventory – Trait subscale
BTLI: Bayesian Tucker–Lewis Index.

## Supplementary material

The supplementary material for this article can be found at http://doi.org/10.1017/S0033291726103821.
