# Supplementary material for: Developmental trauma in functional motor disorder: the mediating roles of affective symptoms and multimorbidity
Source: Psychol Med. 2026 Mar 27;56:e85. doi: 10.1017/S0033291726103821 (PMC13079214; doi:10.1017/S0033291726103821)
Supplement: Sojka et al. supplementary material [file S0033291726103821sup001.docx]

**Supplementary Results**

**Supplementary Table 1** Factor loadings of the Bayesian Structural Equation Model.

| **Latent variable** | **Manifest variable** | **β** | **Posterior SD** | **95% CrI lower** | **95% CrI upper** |
| --- | --- | --- | --- | --- | --- |
| **CTQ** | **EA** | 0.90 | 0.05 | 0.81 | 1.00 |
|  | **EN** | 0.83 | 0.05 | 0.74 | 0.93 |
|  | **PA** | 0.72 | 0.05 | 0.62 | 0.82 |
|  | **PN** | 0.72 | 0.05 | 0.62 | 0.83 |
|  | **SA** | 0.44 | 0.06 | 0.33 | 0.56 |
| **FMD** | **QPC** | 0.52 | 0.05 | 0.44 | 0.61 |
|  | **SMSS** | 0.49 | 0.06 | 0.39 | 0.60 |
|  | **FSS** | 0.44 | 0.05 | 0.34 | 0.54 |
|  | **PAIN** | 0.35 | 0.05 | 0.25 | 0.45 |
| **DepAnx** | **BDI-II** | 0.89 | 0.04 | 0.82 | 0.97 |
|  | **STAI-Y2** | 0.75 | 0.04 | 0.68 | 0.84 |

**Supplementary Table 2** Descriptive statistics.

| **Variable** | **HC** | **FMD** | **Difference (FMD−HC) [95% CrI]** | **BF_10_** |
| --- | --- | --- | --- | --- |
| **Females, n (%)** | 159 (74.0) | 237 (73.6) | -0.02 [-0.41,0.38]* | 0.1 |
| **AGE, mean (SD)** | 44.65 (12.24) | 48.75 (12.42) | 4.05 [1.92, 6.18] | 90.19 |
| **SMSS, mean (SD)** | 0.32 (0.80) | 5.97 (2.55) | 5.65 [5.28, 6.01] | 2.53E+107 |
| **BDI-II, mean (SD)** | 7.32 (8.14) | 19.87 (12.57) | 12.43 [10.54, 14.33] | 1.35E+30 |
| **STAI-Y2, mean (SD)** | 38.71 (10.01) | 49.52 (11.30) | 10.71 [8.84, 12.60] | 5.01E+23 |
| **Pain, mean (SD)** | 2.09 (2.00) | 5.98 (2.30) | 3.90 [3.51, 4.27] | 2.01E+64 |
| **FSS, mean (SD)** | 3.61 (1.41) | 5.57 (1.31) | 1.96 [1.73, 2.19] | 7.98E+45 |
| **QPC, mean (SD)** | 1.54 (1.91) | 4.71 (3.00) | 3.17 [2.72, 3.62] | 4.10E+33 |
| **CTQ** |  |  |  |  |
| **EA, mean (SD)** | 8.26 (4.28) | 9.73 (5.14) | 1.46 [0.63, 2.31] | 31.17 |
| **PA, mean (SD)** | 5.78 (2.00) | 7.02 (3.75) | 1.23 [0.69, 1.78] | 1133.26 |
| **SA, mean (SD)** | 5.46 (1.80) | 6.28 (3.41) | 0.81 [0.32, 1.31] | 14.98 |
| **EN, mean (SD)** | 11.25 (4.89) | 12.50 (5.66) | 1.26 [0.35, 2.18] | 2.99 |
| **PN, mean (SD)** | 6.81 (2.77) | 7.86 (3.48) | 1.04 [0.50, 1.60] | 70.57 |
| **EA, N above cut-off (%)** | 59 (27) | 133 (41) |  |  |
| **PA, N above cut-off (%)** | 22 (10) | 78 (24) |  |  |
| **SA, N above cut-off (%)** | 13 (6) | 46 (14) |  |  |
| **EN, N above cut-off (%)** | 56 (26) | 115 (36) |  |  |
| **PN, N above cut-off (%)** | 58 (27) | 134 (42) |  |  |
| **At least one above cut-off (%)** | 97 (45) | 200 (62) |  |  |
| **Phenotype** |  |  |  |  |
| **Gait, n (%)** |  | 103 (32) |  |  |
| **Tremor, n (%)** |  | 83 (25.8) |  |  |
| **Weakness, n (%)** |  | 77 (23.9) |  |  |
| **Dystonia, n (%)** |  | 41 (12.7) |  |  |
| **Jerks, n (%)** |  | 18 (5.6) |  |  |

Values are mean (SD) unless stated. *log odds-ratio; SMSS = Subjective Motor Symptom Severity. BDI-II = Beck Depression Inventory-II. STAI-Y2 = State–Trait Anxiety Inventory, Trait (Form Y-2). FSS = Fatigue Severity Scale. QPC= Cognitive Complaints Questionnaire. CTQ subscales: EA = emotional abuse; PA = physical abuse; SA = sexual abuse; EN = emotional neglect; PN = physical neglect. Differences shown as posterior mean [95% CrI]; BF_10_ is the Bayes Factor for the alternative hypothesis, that group difference is non-zero. ​​BF₁₀ > 1 indicates evidence supporting the alternative hypothesis, whereas BF₁₀ < 1 favours the null. Interpretation followed standard guidelines (e.g., BF₁₀ ≈ 1: negligible; 3–10: moderate; >10: strong evidence). Clinical cut-off values for the presence of traumatic experience is based on [(Walker et al., 1999)](https://paperpile.com/c/zjOe98/xJxP).

**Supplementary Table 3** Mean values and 95% credible intervals for regression coefficients.

| **Outcome** | **predictor** | **β [95% CrI]** | | |
| --- | --- | --- | --- | --- |
|  |  | **FMD** | **HC** | **FMD−HC** |
| **SMSS** | EA | 0.15 [0.04, 0.25] | 0.00 [-0.14, 0.14] | 0.14 [-0.03, 0.32] |
| **SMSS** | EN | -0.00 [-0.11, 0.11] | 0.00 [-0.11, 0.12] | -0.01 [-0.16, 0.15] |
| **SMSS** | PA | -0.01 [-0.10, 0.07] | 0.01 [-0.15, 0.17] | -0.02 [-0.20, 0.15] |
| **SMSS** | PN | 0.05 [-0.04, 0.14] | 0.03 [-0.09, 0.14] | 0.02 [-0.12, 0.17] |
| **SMSS** | SA | 0.00 [-0.07, 0.07] | 0.04 [-0.10, 0.18] | -0.04 [-0.20, 0.11] |
| **BDI-II** | EA | 0.37 [0.22, 0.51] | 0.02 [-0.17, 0.21] | 0.34 [0.10, 0.58] |
| **BDI-II** | EN | 0.13 [-0.01, 0.27] | 0.13 [-0.03, 0.29] | -0.00 [-0.22, 0.22] |
| **BDI-II** | PA | -0.06 [-0.17, 0.05] | -0.07 [-0.29, 0.14] | 0.01 [-0.23, 0.26] |
| **BDI-II** | PN | 0.01 [-0.12, 0.13] | 0.04 [-0.12, 0.20] | -0.03 [-0.23, 0.17] |
| **BDI-II** | SA | 0.00 [-0.09, 0.09] | 0.14 [-0.05, 0.34] | -0.14 [-0.35, 0.08] |
| **STAI-Y2** | EA | 0.32 [0.16, 0.47] | -0.03 [-0.25, 0.18] | 0.35 [0.09, 0.61] |
| **STAI-Y2** | EN | 0.19 [0.04, 0.34] | 0.26 [0.08, 0.44] | -0.07 [-0.30, 0.17] |
| **STAI-Y2** | PA | -0.10 [-0.22, 0.02] | -0.08 [-0.32, 0.16] | -0.02 [-0.29, 0.25] |
| **STAI-Y2** | PN | -0.05 [-0.18, 0.08] | 0.04 [-0.13, 0.22] | -0.10 [-0.31, 0.12] |
| **STAI-Y2** | SA | -0.03 [-0.13, 0.06] | 0.09 [-0.12, 0.30] | -0.12 [-0.35, 0.11] |
| **Pain** | EA | 0.06 [-0.08, 0.19] | 0.09 [-0.09, 0.28] | -0.04 [-0.26, 0.19] |
| **Pain** | EN | 0.02 [-0.11, 0.16] | -0.02 [-0.17, 0.14] | 0.04 [-0.16, 0.25] |
| **Pain** | PA | -0.05 [-0.16, 0.06] | -0.01 [-0.22, 0.19] | -0.04 [-0.27, 0.19] |
| **Pain** | PN | 0.08 [-0.04, 0.19] | -0.07 [-0.22, 0.08] | 0.15 [-0.04, 0.33] |
| **Pain** | SA | 0.00 [-0.08, 0.09] | 0.34 [0.16, 0.52] | -0.34 [-0.53, -0.14] |
| **FSS** | EA | 0.17 [0.03, 0.32] | 0.03 [-0.17, 0.23] | 0.14 [-0.11, 0.39] |
| **FSS** | EN | 0.03 [-0.11, 0.17] | 0.16 [-0.01, 0.33] | -0.13 [-0.36, 0.09] |
| **FSS** | PA | -0.13 [-0.24, -0.01] | -0.25 [-0.48, -0.02] | 0.12 [-0.13, 0.38] |
| **FSS** | PN | -0.00 [-0.12, 0.12] | 0.05 [-0.12, 0.22] | -0.05 [-0.26, 0.15] |
| **FSS** | SA | 0.01 [-0.08, 0.10] | 0.22 [0.03, 0.42] | -0.21 [-0.43, 0.00] |
| **QPC** | EA | 0.27 [0.11, 0.42] | -0.01 [-0.22, 0.19] | 0.28 [0.02, 0.54] |
| **QPC** | EN | 0.00 [-0.15, 0.15] | 0.11 [-0.07, 0.28] | -0.11 [-0.34, 0.13] |
| **QPC** | PA | -0.03 [-0.14, 0.09] | 0.11 [-0.13, 0.34] | -0.13 [-0.40, 0.13] |
| **QPC** | PN | 0.07 [-0.06, 0.21] | -0.07 [-0.24, 0.10] | 0.14 [-0.07, 0.36] |
| **QPC** | SA | -0.02 [-0.11, 0.08] | 0.15 [-0.05, 0.36] | -0.17 [-0.40, 0.06] |

**Supplementary Table 4A**

Regression coefficients of mediation model without MMi

| **Outcome** | **Predictor** | **β** | **Posterior SD** | **95% CrI**  **lower** | **95%CrI**  **upper** |
| --- | --- | --- | --- | --- | --- |
| DepAnx | CTQ | 0.51 | 0.07 | 0.38 | 0.64 |
| FMD | CTQ | 0.04 | 0.09 | -0.14 | 0.20 |
| FMD | DepAnx | 0.82 | 0.09 | 0.62 | 1.03 |
| FMD (indirect effect) | CTQ -> DepAnx | 0.42 | 0.07 | 0.27 | 0.56 |
| FMD (total effect) | CTQ -> DepAnx + CTQ | 0.45 | 0.10 | 0.25 | 0.65 |

**Supplementary Table 4B**

Regression coefficients of the mediation model with FMD factor including only Subjective Motor Symptom Severity (SMSS)

| **Outcome** | **Predictor** | **β** | **Posterior SD** | **95% CrI**  **lower** | **95%CrI**  **upper** |
| --- | --- | --- | --- | --- | --- |
| DepAnx | CTQ | 0.51 | 0.07 | 0.38 | 0.64 |
| FMD | CTQ | 0.04 | 0.09 | -0.14 | 0.20 |
| FMD | DepAnx | 0.36 | 0.07 | 0.23 | 0.49 |
| FMD (indirect effect) | CTQ -> DepAnx | 0.18 | 0.04 | 0.10 | 0.26 |
| FMD (total effect) | CTQ -> DepAnx + CTQ | 0.23 | 0.07 | 0.10 | 0.36 |

**Supplementary Table 5**

Regression coefficients of the reverse mediation model without MMi

| **Outcome** | **Predictor** | **β** | **Posterior SD** | **95% CrI**  **lower** | **95%CrI**  **upper** |
| --- | --- | --- | --- | --- | --- |
| DepAnx | CTQ | 0.37 | 0.08 | 0.22 | 0.52 |
| DepAnx | FMD | 0.83 | 0.11 | 0.63 | 1.05 |
| FMD | DepAnx | 0.35 | 0.08 | 0.20 | 0.50 |
| DepAnx (indirect effect) | CTQ -> FMD | 0.31 | 0.08 | 0.17 | 0.45 |
| DepAnx (total effect) | CTQ -> FMD + CTQ | 0.66 | 0.09 | 0.47 | 0.84 |

**Supplementary Table 6A**

Regression coefficients of the mediation model with MMi

| **Outcome** | **Predictor** | **β** | **Posterior SD** | **95% CrI**  **lower** | **95%CrI**  **upper** |
| --- | --- | --- | --- | --- | --- |
| MMi | CTQ | 0.13 | 0.06 | 0.00 | 0.25 |
| DepAnx | CTQ | 0.49 | 0.07 | 0.36 | 0.63 |
| DepAnx | MMi | 0.24 | 0.06 | 0.12 | 0.37 |
| FMD | CTQ | 0.03 | 0.09 | -0.14 | 0.21 |
| FMD | DepAnx | 0.77 | 0.10 | 0.58 | 0.98 |
| FMD | MMi | 0.24 | 0.09 | 0.07 | 0.42 |
| FMD (indirect effect) | CTQ -> DepAnx | 0.38 | 0.07 | 0.25 | 0.51 |
| FMD (indirect effect) | CTQ ->MMi -> DepAnx | 0.05 | 0.03 | 0.00 | 0.10 |
| FMD (indirect effect) | CTQ -> MMi | 0.03 | 0.02 | -0.01 | 0.07 |
| FMD (indirect effect) | MMi -> DepAnx | 0.18 | 0.05 | 0.08 | 0.29 |
| FMD (total effect) | CTQ -> DepAnx + CTQ | 0.41 | 0.10 | 0.21 | 0.61 |

**Supplementary Table 6B**

Regression coefficients from the multimorbidity (MMi) mediation model including only somatic and neurological conditions (psychiatric diagnoses excluded).

| **Outcome** | **Predictor** | **β** | **Posterior SD** | **95% CrI**  **lower** | **95%CrI**  **upper** |
| --- | --- | --- | --- | --- | --- |
| MMi | CTQ | 0.09 | 0.07 | -0.04 | 0.22 |
| DepAnx | CTQ | 0.50 | 0.07 | 0.37 | 0.64 |
| DepAnx | MMi | 0.13 | 0.06 | 0.01 | 0.25 |
| FMD | CTQ | 0.03 | 0.09 | -0.15 | 0.20 |
| FMD | DepAnx | 0.81 | 0.11 | 0.62 | 1.05 |
| FMD | MMi | 0.26 | 0.09 | 0.10 | 0.43 |
| FMD (indirect effect) | CTQ -> DepAnx | 0.41 | 0.07 | 0.26 | 0.55 |
| FMD (indirect effect) | CTQ ->MMi -> DepAnx | 0.04 | 0.03 | -0.02 | 0.09 |
| FMD (indirect effect) | CTQ -> MMi | 0.02 | 0.02 | -0.02 | 0.06 |
| FMD (indirect effect) | MMi -> DepAnx | 0.11 | 0.05 | 0.06 | 0.21 |
| FMD (total effect) | CTQ -> DepAnx + CTQ | 0.44 | 0.10 | 0.24 | 0.64 |

Walker EA, Unutzer J, Rutter C, Gelfand A, Saunders K, VonKorff M, Koss MP, Katon W. Costs of health care use by women HMO members with a history of childhood abuse and neglect. Arch Gen Psychiatry. 1999 Jul;56(7):609-13. doi: 10.1001/archpsyc.56.7.609. PMID: 10401506.
